# Clinical features and phylogenetic analysis of Coxsackievirus A9 in Northern Taiwan in 2011

**DOI:** 10.1186/1471-2334-13-33

**Published:** 2013-01-24

**Authors:** Yi-Chuan Huang, Ying-Hsia Chu, Ting-Yu Yen, Wen-Chan Huang, Li-Min Huang, Ai-Ling Cheng, Hurng-Yi Wang, Luan-Yin Chang

**Affiliations:** 1 Division of Pediatric Infectious Disease, Department of Pediatrics, Kaohsiung Chang Gung Memorial Hospital and Chang Gung University College of Medicine, Kaohsiung, Taiwan; 2 Department of Medicine, College of Medicine, National Taiwan University, Taipei, Taiwan; 3 Department of Pediatrics, China Medical University Hospital, China Medical University, Taichung, Taiwan; 4 Department of Pediatrics, National Taiwan University Hospital, College of Medicine, National Taiwan University, No. 8 Chung-Shan South Road, Taipei, 10002, Taiwan; 5 Institute of Clinical Medicine, College of Medicine, National Taiwan University, Taipei, Taiwan

**Keywords:** Coxsackievirus A9, Enterovirus, Viral exanthema, Phylogenetic tree

## Abstract

**Background:**

Coxsackievirus A9 (CA9) was one of the most prevalent serotype of enteroviral infections in Taiwan in 2011. After several patient series were reported in the 1960s and 1970s, few studies have focused on the clinical manifestations of CA9 infections. Our study explores and deepens the current understanding of CA9.

**Methods:**

We analyzed the clinical presentations of 100 culture-proven CA9-infected patients in 2011 by reviewing their medical records and depicted the CA9 phylogenetic tree.

**Results:**

Of the 100 patients with culture-proven CA9 infections, the mean (SD) age was 4.6 (3.4) years and the male to female ratio was 1.9. For clinical manifestations, 96 patients (96%) had fever and the mean (SD) duration of fever was 5.9 (3.4) days. Sixty one patients (61%) developed a skin rash, and the predominant pattern was a generalized non-itchy maculopapular rash without vesicular changes. While most patients showed injected throat, oral ulcers were found in only 19 cases (19%), among whom, 6 were diagnosed as herpangina. Complicated cases included: aseptic meningitis (n=8), bronchopneumonia (n=6), acute cerebellitis (n=1), and polio-like syndrome (n=1). Phylogenetic analysis for current CA9 strains is closest to the CA9 isolate 27-YN-2008 from the border area of mainland China and Myanmar.

**Conclusions:**

The most common feature of CA9 during the 2011 epidemic in Taiwan is generalized febrile exanthema rather than herpangina or hand, foot, and mouth disease. Given that prolonged fever and some complications are possible, caution should be advised in assessing patients as well as in predicting the clinical course.

## Background

Human enteroviruses (HEVs) are RNA viruses consisting of polioviruses, coxsackie A viruses, coxsackie B viruses, echoviruses, and enterovirus 68–71 under the traditional pathogenesis-based taxonomy. Since the 1990s, viral genome analysis has brought about new classifications, HEV-A, HEV-B, HEV-C, and HEV-D, based on the nucleotide sequence of the VP1 region and it was accepted by International Committee on Taxonomy of Viruses (ICTV) in the mid of year 2000 [1]. Coxsackievirus A9 (CA9) used to belong to coxsackie A viruses and has been reclassified into the HEV-B group [1].

In Taiwan there are annual spring and summer HEV epidemics, with children under age 5 constituting the majority of patients. Clinical manifestations may include herpangina and hand, foot, -and mouth disease (HFMD). In 2011, however, a fair portion of the 3,308 non-polio HEV infections reported by Taiwan’s Center for Disease Control (CDC) did not show herpangina or HFMD, but presented with fever and a somewhat characteristic exanthema different from HFMD; CA9 was isolated in 499 of these cases [2].

In terms of CA9-related skin rashes, Lerner et al. summarized what had been observed in 39 patients as a 2 to 13 mm maculopapular exanthema beginning on the face or trunk during fever and sometimes spreading to the extremities [3,4]. In another 20 cases studied by Cherry et al., vesicular, urticarial, or petechial rashes have also been reported [5]. Although these observations have been made, renewed research efforts on an expanded number of patients are still needed to shed light on both the rash morphology and also other systemic and local manifestations of CA9 infections. Such understandings may contribute to accurate diagnosis and course prediction that help ease parental worries and avoid serious complications, including pneumonitis, pericarditis, myocarditis, and central nervous inflammation with paralytic sequelae, which have all been seen, though rarely, in literature [6,7].

Our purpose is to investigate the clinical features of CA9 infections based on the 2011 Taiwan epidemic. We also constructed a viral phylogenetic tree in order to understand the molecular epidemiology of CA9 infections in Northern Taiwan.

## Methods

### Case definition

In this study, we collected data from 100 patients with laboratory confirmed CA9 infections at National Taiwan University Hospital in 2011. Throat swabs were taken at clinics and wards from suspected cases for viral cul-tures and reverse-transcriptase polymerase chain reaction (RT-PCR) followed by VP1 gene sequencing.

### Research design and data collection

We reviewed the medical records of confirmed CA9 cases. While studying each patient’s medical chart review or in-clinic examination, we completed a checklist which included the patient profile data and common viral symptoms (headache, decreased activity, sore throat, rhinorrhea, cough, and gastrointestinal symptoms such as vomiting and diarrhea). Special attention was paid to fever (including length of febrile period and highest temperature) and rash pattern (including temporal relation to fever, distribution, and morphology) as fever and rash were the two most common complaints in our cases and usually aroused considerable parental worry. Lastly we made special references to conjunctivitis, which was often presented by children affected by the concurrent adenovirus epidemic in Taiwan, and perioral/oral lesions, which had been characteristic in our previous experience of coxsackieviral infections.

This study was approved by the Institutional Review Board of National Taiwan University Hospital and the approval number was 201007053R. Informed consent for participation in the study and the publication of the images was obtained from the parents of the enrolled children.

### Viral identification, serotyping and phylogenetic analysis

The throat swab was inoculated into cell lines MRC-S, LLC-MK2, HEp-2, and human rhabdomyosarcoma cells. When the enteroviral cytopathic effect exceeded 50%, cells were scraped and enterovirus was confirmed via indirect immunofluorescence assay with panenteroviral antibody (Chemicon International, Inc., Temecula, CA, USA). Viral RNA was extracted from throat swabs using QIAamp Viral RNA Mini Kit (Qiagen, Santa Clara, CA). The partial VP1 gene was amplified by RT-PCR as described previously [8], the VP1 amplicon was 316 bp (VP1 nucleotide position from 97 to 412 based on the CA9 GenBank reference sequence GQ294574) and it was then sequenced using BigDye Terminator Ready Reaction Cycle Sequencing Kit and sequencer ABI 3730 (Applied Biosystems, Foster City, CA, USA). The serotype of enteroviruses was inferred by comparison of the partial VP1 sequence to those in the public gene database containing VP1 sequences for the strains of all the 67 human enterovirus serotypes, and CA9 was defined when the partial VP1 amino acid sequence showed ≧88% homology to the VP1 amino acid sequence in CA9 prototype strains [8]. We selected the first sequence from every eight consecutive cases from current study and 13 sequences were selected to build a phylogenetic tree. With the 13 newly obtained sequences (GenBank accession numbers: KC286622, KC286623, KC286624, KC286625, KC286626, KC286627, KC286628, KC286629, KC286630, KC286631, KC286632, KC286633, KC286634), 19 formerly reported CA9 sequences (GenBank accession numbers: HQ844647, GQ294574, HM752812 , HQ897682, FJ525948, GQ329731, GQ246517, AB167980, GQ329729, GQ329728, AB268124, AB268124, AY573578, AB268121, AB268123, GQ329730, FJ868282, GU142874, AM236967) and 1 CA16 sequence (GenBank accession number JX473437), used as an outgroup, in the NCBI GenBank database, we constructed a phylogenetic tree based on the 280 bp of VP1 region in MEGA software version 4.1 using the neighbor-joining method and bootstrap analysis with 1,000 replications [9].

## Results

### Seasonal distribution and demography

In 2011, a total of 100 CA9 cases with available medical records were included in our study. The number of CA9 infections peaked in June, 2011, with 59 cases detected within one month, and began to drop in July. The majority of CA9-infected patients in our study were aged 2 to 8 years old with mean age 4.6 ± 3.4 years old (Table 1). The eldest patient was a 41-year-old male and the youngest was a 5-day-old infant. The male-to-female ratio was 1.9:1.

**Table 1:** Demographic and clinical characteristics of 100 CA9-infected patients

**Characteristic**	**CA9 patients**
Age (years)	4.6 (3.4)*
Male/female (ratio)	65/35 (1.9)
Fever reported	96 (96%)
Duration of Fever in 58 cases (days)	5.9 (3.4)*
Skin rash	61 (61%)
Cough	33 (33%)
Rhinorrhea	31 (31%)
Sore throat	12 (12%)
Diarrhea	10 (10%)
Vomiting	21 (21%)
Headache	27 (27%)
Dyspnea	7 (7%)
Decreased activity	26 (26%)
Oral ulcer	19 (19%)
Exudative tonsils	10 (10%)
Conjunctivitis	9 (9%)

*Data are expressed as mean (SD).

### Clinical manifestations

Ninety six patients (96%) were reported experiencing fever; among them the caregivers of 55 patients confirmed taking tympanic temperatures ≧ 38.5°C. The average reported tympanic temperature was 39.1°C. The highest temperature taken in our outpatient clinics was 40.6°C, higher than all reported measurements. For the 58 patients with available data, the average fever duration was 5.9 days (Table 1) and the longest fever duration was 16 days.

CA9-infected patients sometimes experienced fever and symptoms of upper respiratory tract infection, defined as rhinorrhea, cough, and/or sore throat (Table 1). Cough and rhinorrhea each occurred in about 30% of patients, sore throat in 12%, vomiting in 21%, diarrhea in 10%, conjunctivitis in 9%, and exudative tonsils in 10%. About 26% of patients had a decrease in activity noted by caregivers.

### Characteristics of skin rash

**Figure 1:**
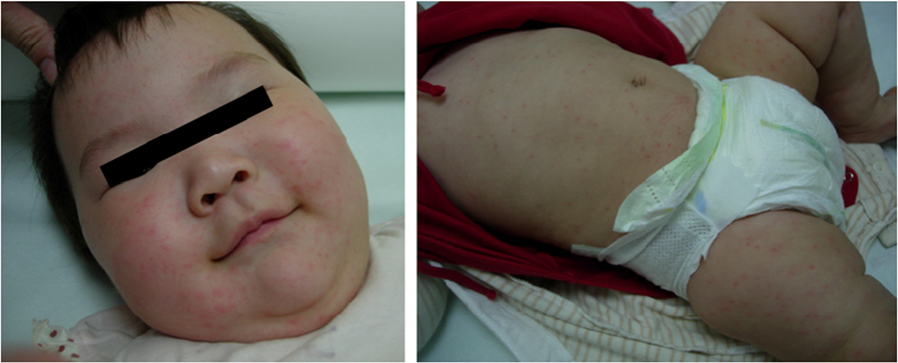
**Typical skin rash observed in patients with CA9 infection.** The most frequently observed pattern was generalized maculopapular without vesicular change. Face, trunk, and extremities are all commonly involved.

Sixty one percent of CA9-infected patients had skin rashes observed during physical examination (Table 1). The most common rash pattern was generalized maculopapular rash (size from 1 to 3 mm) extending to the face, trunk, and extremities as shown in Figure 1. Only 5% of CA9 rash patients had vesicular rashes (Table 2), which appeared as 1 to 3 mm vesicles dispersed on a maculopapular background. Hands and/or feet were involved in 10% of cases. In rare cases we also noted local or generalized morphology variants as morbilliform, scalatiniform (sand paper-like), urticarial with pruritus, and nonblanchable petechiae. In 51% of rash cases, the exanthema was reported to appear after the first fever episode when the patient was still occasionally febrile. Three percent of rash cases did not experience fever.

**Table 2:** Characteristics of skin rashes of 61 CA9-infected patients

**Characteristic**	**No. (%)**
**Location involved**	
Face	37 (61%)
Trunk	43 (70%)
Extremities	43 (70%)
Hands and/or feet	6 (10%)
**Morphology**	
Maculopapular	34 (56%)
Papulovesicular	3 (5%)
Morbilliform	2 (3%)
Scalatiniform	3 (5%)
Urticarial	1 (2%)
Petechial	4 (7%)
Maculopapular with petechiae	2 (3%)
Papulovesicular with petechiae	1 (2%)
Rash with morphology not recorded	11 (18%)
**Temporal relation to fever**	
Appeared before fever onset	4 (7%)
Appeared simultaneously with fever onset	6 (10%)
Appeared after fever onset when still febrile	31 (51%)
Febrile rash with temporal order not recorded	18 (30%)
Rash without fever	2 (3%)
**Other skin changes**	
Scaling	1 (2%)
Pruritis	14 (23%)

### Complications

**Figure 2:**
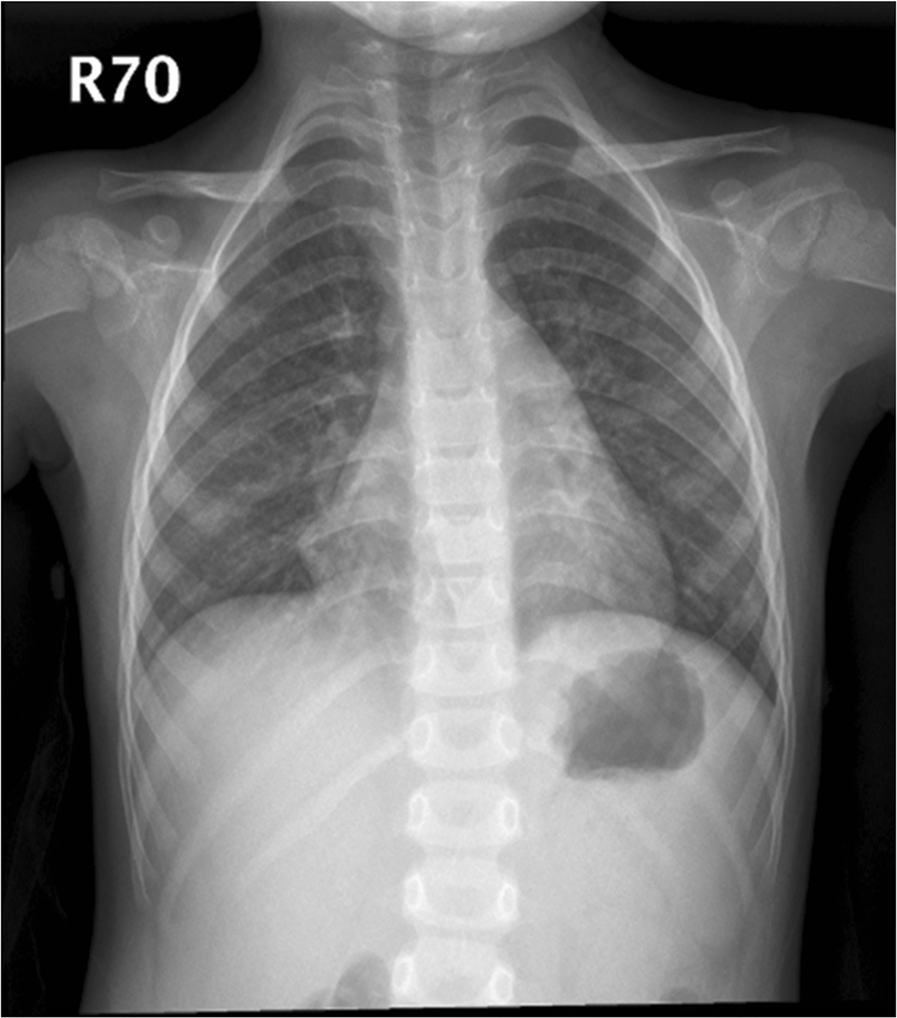
Chest X ray of a 4-year-old, CA9-infected female patient complicated with bronchopneumonia.

Although the majority of patients in this study had complete recoveries, several complicated cases were seen, including aseptic meningitis (n=8), bronchopneumonia (n=6), 1 acute cerebellitis (n=1), and 1 polio-like syndrome (n=1). Severe headache and vomiting episodes were experienced by the 8 cases with aseptic meningitis. All aseptic meningitis cases had complete recoveries without sequelae. The 6 bronchopneumonia cases presented with nonproductive cough. Bilateral rales were noted on chest auscultation, and chest X-ray of 1 of the cases was shown in Figure 2.

The polio-like syndrome case was a 19-month-old boy that had been previously healthy. His illness began with an episode of nonfebrile loose defecation. The next day a limping gait was noted and he was admitted to our hospital. Upon admission, physical and neurological examination findings were unremarkable. Fever occurred with a generalized maculopapular skin rash involving the face, trunk, and extremities on the second day of admission and the fever subsided one day later. Although cervical spine magnetic resonance image (MRI), electroencephalography, and central conduction time from median nerves and tibial nerves were all normal, the patient’s limping gait and left ankle stiffness showed little improvement during his hospitalization. Rehabilitative courses were given after discharge.

The acute cerebellitis case was a 6.5-year-old boy. Two days after a sudden onset of fever and headache, he was found to have difficulty keeping balance when standing or walking, thus he was admitted. Upon admission, both the finger-nose-finger test and the rapid alternating test showed bilateral impairment. Truncal ataxia was also noted. Electroencephalography and brain MRI were normal. The fever subsided on the second day of hospitalization. However, dysmetria and ataxic gait remained evident upon discharge. He is currently in rehabilitative courses.

**Figure 3:**
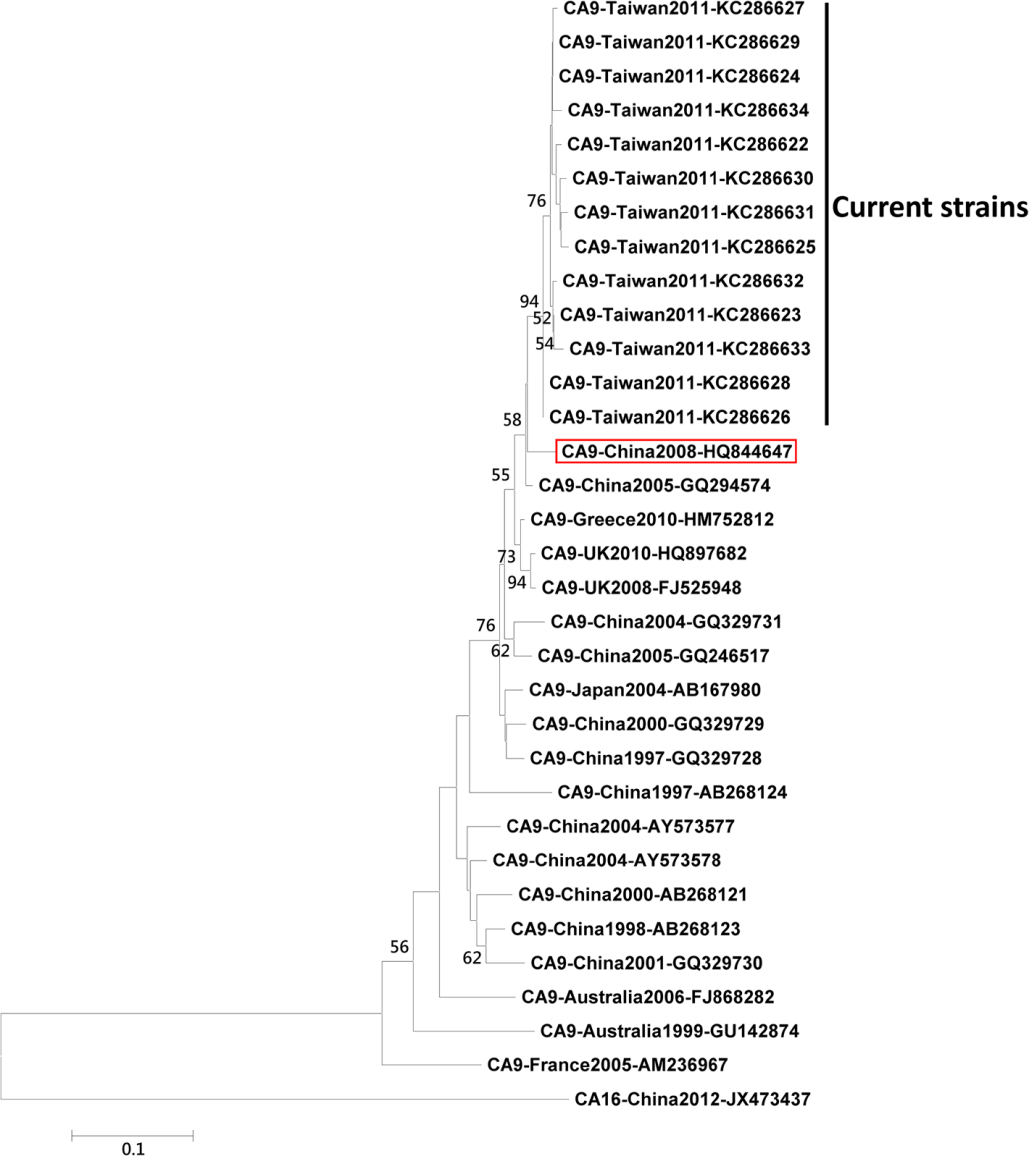
**Phylogenetic tree of coxsackievirus A9 (CA9), based on partial VP1 gene.** In bracket CA9 isolate 27-YN-2008 (CA9-China2008-HQ844647) isolated near the border of Yunnan province, Mainland China and Myanmar in 2008.

The phylogenetic analysis results are showed in Figure 3. All the current CA9 strains from 2011 epidemic were within the same lineage and closest to the CA9 isolate 27-YN-2008 (GenBank accession number HQ844647) which was isolated from one healthy child near the border of Yunnan province of Mainland China with Myanmar in 2008.

## Discussion

Patients with enterovirus infections may present with symptoms varying from an uncomplicated common cold to life-threatening conditions such as encephalitis, myocarditis, and neonatal sepsis [4,6,7]. Most manifestations of enterovirus infections are mild and more than 90% are either asymptomatic or cause a nonspecific febrile illness. The most common presentation on the skin was HFMD. HFMD is a vesicular eruption in the oropharynx, palms, soles, and interdigits of toddlers and school-aged children. Patients often present oral ulcers after 1–2 days of fever and have a characteristic viral exanthem. Lesions are more common on the dorsal surfaces of the hands and feet than in other locations. The most common causative agent is coxsackievirus group A, serotype 16, but strains of enterovirus 71 circulating in East Asia are currently causing outbreaks of HFMD that are associated with a serious rhombencephalitis, with significant mortality [10-13].

In a previous study, CA9 infection was not in the top five enterovirus infections in Taiwan from 1998 to 2006 [10]. In contrast with previous years, the second most common serotype from August 2010 to August 2011 was CA9. In 2011, sixty one (61%) from 100 children infected with CA9 had skin rashes observed during physical examination. The most common rash pattern (56%) was generalized maculopapular rash (size from 1 to 3 mm). Only 4 patients had the rash pattern with papulovesicles with or without petechiae. Of the CA9-infected patients with skin rashes, distribution was mainly seen over the trunk, extremities, face (70%, 70%, and 61%, respectively), while hands and/or feet only 10%; 23% of patients with skin rashes had pruritus. Only 19 patients (19%) had oral ulcers. Our study found that the patients with CA9 infections presented with more widespread skin lesions. Extensive skin involvement in our study indicated a broad spectrum of direct cell infection by the CA9 virus. This finding is consistent with previous studies [3-5,11]. In 51% of rash cases, the exanthem was reported to appear after the first fever episode.

Ninety six percent of the CA9-infected patients were reported to experience fever for a mean duration of 5.9 days and the longest fever duration in the present study was 16 days. In a previous study, the patients with enteroviral infections who had a fever lasting more than three days usually had complications, especially central nervous system involvement [12]. The longer duration of fever may reflect that the CA9 is a more virulent virus associated with more widespread of skin invasion.

CA9 is one of the enteroviruses which may be associated with aseptic meningitis [13,14]. Most enteroviruses infections are asymptomatic, but they can cause a wide variety of clinical diseases, the most common complicated enteroviral infection in pediatric patients remains aseptic meningitis. Serotypes of the HEV-B species, including coxsackievirus B and echovirus, accounted for more than 90% of aseptic meningitis, while coxsackievirus A accounted for less than 5% [15-19]. That could cause sporadic cases, outbreaks, or epidemics of aseptic meningitis worldwide. In the present study, the majority of CA9-infected patients recovered completely. Several complicated cases were seen including aseptic meningitis (n=8), bronchopneumonia (n=6), acute cerebellitis (n=1), and -polio-like syndrome (n=1). As previous studies reported, the most common etiology of aseptic meningitis is enterovirus and the clinical course is benign and self-limited and resolves in 1–2 weeks [20,21]. All our aseptic meningitis patients recovered from the acute illnesses with had no need for intensive care and no long-term sequelae.

HEV-B frequently causes nonspecific illness, including fever, viral exanthem, upper respiratory tract infections, and lower respiratory tract infections (LRI). Bronchopneumonia and bronchiolitis were the most common diagnoses in LRI. Echovirus 4, coxsackievirus B4, and CA9 are the most common serotypes related to LRI [22]. In our 6 CA9-infected patients complicated with bronchopneumonia, they all presented with nonproductive cough and bilateral rales on auscultation. They recovered completely under symptomatic treatment and did not have long-term sequelae.

The genetic heterogeneity of enteroviruses makes it difficult to develop molecular typing that is both reliable and easy to perform. The genome of enteroviruses encodes 11 proteins, including VP1 to VP4, 2A to 2C, and 3A to 3D. Among these genes, VP1 is the one that can induce a human neutralizing antibody response and correlates well with serotypes. The genomic sequence encoding the VP1 capsid protein gives excellent results because of the high correlation between serotype and genetic information, and the availability of a complete database of reference strains isolated in the 1950’s and 1960’s [15]. In this study, the pathogen was identified as CA9 based on the VP1 gene. The CA9 strain circulating in 2011 is most close to the CA9 isolate 27-YN-2008 which was isolated from one healthy child near the border of Yunnan province of Mainland China with Myanmar in 2008.

There are several limitations in this study. The data was obtained retrospectively from northern Taiwan which limits the patient number and the results might not be representative for CA9 infection for all of Taiwan. Further island-wide studies will be conducted until the virus detection method becomes more uniform and accurate. In addition, the CA9 isolated in northern Taiwan is genetically similar to that detected near the Mainland China and Myanmar border. This could be due to close geographic relationship and frequent transportation between the two areas. However, the true transmission route had not been clarified and we did not have clear evidence of that the current strains were imported from Mainland China or Myanmar.

## Conclusion

Patients infected with CA9 during the 2011 epidemic in northern Taiwan commonly presented with generalized febrile exanthema rather than herpangina or HFMD. The CA9 isolated in northern Taiwan is genetically similar to that detected near the border of Mainland China and Myanmar. Although most patients recovered without major complications, a longer febrile period may be a sign of severe infection and careful attention should be paid.

## Competing interests

All the authors declare that they have no competing interests.

## Authors’ contributions

The corresponding author, Dr. Luan-Yin Chang, designed and conducted this clinical study, and revised this manuscript. The first authors, Dr. Ying-Hsia Chu and Dr. Yi-Chuan Huang, enrolled the cases, performed the analyses, and wrote the manuscript. Dr. Ting-Yun Yen, Dr. Wen-Chan Huang, and Dr. Li-Min Huang collected cases and revised the manuscript. MS. Ai-Ling Cheng did the RT-PCR, VP1 sequencing and the phylogenetic tree construction. Dr. Hurng-Yi Wang participated in the phylogenetic tree construction and analysis. All authors have read and approved the final manuscript.

## Pre-publication history

The pre-publication history for this paper can be accessed here:

http://www.biomedcentral.com/1471-2334/13/33/prepub
